# Severity matters: reframing cardiac surgery-associated acute kidney injury

**DOI:** 10.62675/2965-2774.20260115

**Published:** 2026-07-24

**Authors:** Aline Paula Miozzo, Pedro Henrique Rigotti Soares, Denise Battaglini

**Affiliations:** 1 Hospital Moinhos de Vento Porto Alegre RS Brazil Project Office, Hospital Moinhos de Vento - Porto Alegre (RS), Brazil.; 2 Hospital Nossa Senhora da Conceição Grupo Hospitalar Conceição Intensive Care Unit Porto Alegre RS Brazil Intensive Care Unit, Hospital Nossa Senhora da Conceição, Grupo Hospitalar Conceição - Porto Alegre (RS), Brazil.; 3 Universidade do Vale do Rio dos Sinos School of Medicine Department of Internal Medicine Porto Alegre RS Brazil Department of Internal Medicine, School of Medicine, Universidade do Vale do Rio dos Sinos - Porto Alegre (RS), Brazil.; 4 University of Genoa Department of Surgical Sciences and Integrated Diagnostics Genoa Italy Department of Surgical Sciences and Integrated Diagnostics, University of Genoa - Genoa, Italy.; 5 IRCCS Azienda Ospedaliera Metropolitana Anesthesia and Intensive Care Genoa Italy Anesthesia and Intensive Care, IRCCS Azienda Ospedaliera Metropolitana - Genoa, Italy.

## CLINICAL RELEVANCE OF CARDIAC SURGERY-ASSOCIATED ACUTE KIDNEY INJURY

One of the most common complications of cardiac surgery is cardiac surgery-associated acute kidney injury (CSA-AKI), which affects approximately 20 - 30% of patients.^(1)^ Even mild increases in serum creatinine are independently associated with worsened outcomes such as prolonged mechanical ventilation, longer intensive care and hospital stays, higher healthcare costs, and increased short-term mortality.^(2,3)^

Even if patients with CSA-AKI recover renal function before discharge, they remain at increased risk of adverse outcomes,^(2,4)^ and long-term complications, including reduced long-term survival, increased cardiovascular morbidity, and chronic kidney disease (CKD). The need for renal replacement therapy, despite being uncommon, has a disproportionately high death rate in these patients.

Pathophysiologically, CSA-AKI results from intricate interactions among perioperative hemodynamic instability, oxidative stress, hemolysis, systemic inflammation, and ischemia-reperfusion injury. Renal sensitivity is also increased by fluid variations, aortic cross-clamping, cardiopulmonary bypass, and exposure to nephrotoxic agents.^(5)^ Exposure to nephrotoxic agents may further deteriorate renal function and fluid variations, which can result in venous congestion or hypoperfusion.^(6)^ Hypovolemia, vasoplegia, and blood pressure fluctuations are manifestations of perioperative hemodynamic instability that impairs renal perfusion and increases the risk of damage.^(6)^

Cardiac surgery-associated acute kidney injury is a possible cause of organ dysfunction as well as a marker of overall perioperative stress. Therefore, improving outcomes for patients undergoing cardiac surgery requires early risk assessment, preventive measures, and strict management.^(6)^

## MAIN FINDINGS OF THE STUDY

The study by Prabhakar et al.^(7)^ conducted within a single multi-hospital healthcare system offers a contemporary and robust real-world assessment of the incidence and clinical impact of CSA-AKI. In this large retrospective observational cohort of adult cardiac surgery intensive care unit (ICU) patients, approximately one in six developed CSA-AKI, with stage 1 accounting for the majority of cases, and stages 2 and 3 occurring less frequently but carrying greater clinical consequences. The occurrence of AKI was more common among patients with higher baseline renal risk, diabetes, and those exposed to perioperative factors such as contrast studies, nephrotoxic antibiotics, and blood product transfusions.

Beyond its incidence, the study clearly demonstrates that CSA-AKI is strongly associated with adverse postoperative trajectories. Patients who developed AKI required significantly prolonged mechanical ventilation and experienced longer ICU and hospital lengths of stay. Notably, more than one quarter of affected patients failed to achieve complete renal recovery by discharge, underscoring the persistence of organ dysfunction beyond the immediate perioperative period. Most strikingly, a marked severity-dependent gradient was observed for 30-day mortality, with dramatically increased odds among patients with stage 2 and stage 3 AKI.

**Figure 1:**
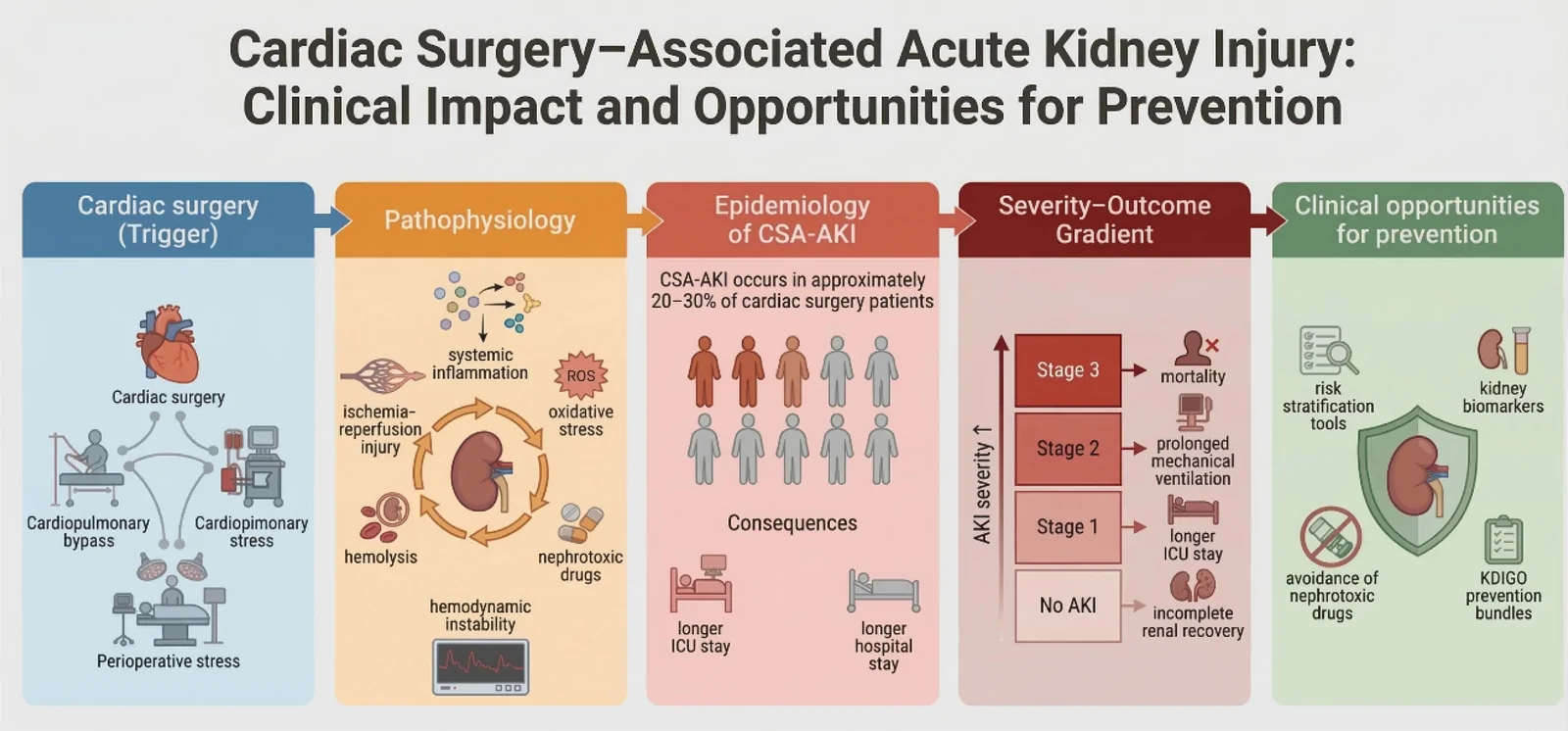
Conceptual overview of cardiac surgery-associated acute kidney injury. The figure was created by the authors with the assistance of the Google Gemini Artificial Intelligence tool.

CSA-AKI - cardiac surgery-associated acute kidney injury; ICU - intensive care unit.

Taken together, these findings reinforce CSA-AKI as a common and clinically consequential complication in contemporary cardiac surgical practice. Consistent with prior large cohort analyses demonstrating a clear dose-response relationship between AKI stage and postoperative mortality.^(7)^ The present study further supports the notion that even incremental increases in AKI severity translate into disproportionately worse clinical outcomes.

## ACUTE KIDNEY INJURY SEVERITY IS THE DOMINANT DRIVER OF OUTCOMES

Building upon these findings, this study further underscores that CSA-AKI severity functions as a dominant determinant of postoperative outcomes rather than merely reflecting baseline vulnerability. Although CKD has long been recognized as a perioperative risk factor, growing evidence indicates that the magnitude of acute renal dysfunction carries an independent and graded prognostic impact. Large prospective investigations, including the epidemiology of surgery associated acute kidney injury (EPIS-AKI) study, demonstrate a consistent association between increasing AKI severity and mortality across surgical populations.^(8)^ In cardiac surgery specifically, subgroup analyses confirm that higher Kidney Disease Improving Global Outcomes (KDIGO) stages confer disproportionately greater risk, independently of baseline characteristics.^(9)^ Moreover, studies in patients with preserved preoperative renal function show that the development and progression of AKI, more than underlying CKD, substantially influence postoperative survival.^(7)^

These findings support a shift in interpretation, as severe AKI should not be viewed solely as a surrogate of comorbidity burden but as an active pathophysiological process within perioperative organ dysfunction. Renal injury likely reflects cumulative hemodynamic instability, inflammatory activation, and microcirculatory impairment, mechanisms closely linked to adverse clinical trajectories. Recognizing AKI as a modifiable driver of harm reinforces the urgency of comprehensive perioperative strategies aimed at its prevention, early detection, and mitigation.

## IMPLICATIONS FOR CLINICAL PRACTICE

From a clinical standpoint, the findings of the present study underscore the need for a structured, proactive perioperative approach for patients undergoing cardiac surgery. In the absence of specific pharmacologic therapies to directly address CSA-AKI, management strategies continue to focus on prevention, early identification, and optimized supportive care. In this regard, a multimodal risk-stratified strategy is crucial, incorporating meticulous intraoperative hemodynamic optimization, goal-directed perfusion during cardiopulmonary bypass, and vigilant postoperative surveillance, particularly for patients at elevated risk. Emerging evidence also indicates that the integration of renal biomarkers may facilitate the early identification of vulnerable individuals and enable the timely implementation of KDIGO-based care bundles, which have demonstrated potential in reducing the incidence and progression of AKI.^(10,11)^

Moreover, the recognition that even mild degrees of postoperative renal dysfunction are associated with adverse clinical outcomes emphasizes the importance of vigilant renal function monitoring and minimizing exposure to nephrotoxic agents throughout the perioperative period. Integrating risk stratification tools, emerging biomarkers, and kidney-protective strategies into perioperative care pathways may thus represent a critical step toward mitigating the clinical impact of CSA-AKI in contemporary cardiac surgical practice.^(12)^

## LIMITATIONS AND FUTURE DIRECTIONS

Despite its important contributions, some limitations warrant consideration. The retrospective design precludes causal inference and may be subject to residual confounding, particularly given the absence of detailed perioperative hemodynamic variables that could further clarify mechanisms of renal injury. In addition, incomplete urine output data may have led to underestimation of AKI incidence and severity, as classification relied predominantly on serum creatinine criteria. The relatively small number of patients in higher KDIGO stages also limits more granular comparisons between severe categories.

Beyond methodological constraints, important questions remain unanswered. The lack of long-term follow-up prevents assessment of progression from AKI to CKD, a clinically meaningful trajectory for patients who fail to achieve full renal recovery. Furthermore, although growing evidence supports biomarker-guided identification of high-risk individuals and implementation of KDIGO-based care bundles, such strategies were not evaluated in the present analysis.

Future prospective studies integrating hemodynamic monitoring, biomarker-guided risk stratification, and standardized preventive protocols will be essential to determine whether early detection, prevention of CSA-AKI development, and targeted interventions can meaningfully reduce its incidence, progression, and downstream consequences. Advancing this field will require not only improved identification of vulnerable patients but also the systematic implementation of perioperative renal-protective strategies to mitigate hemodynamic and inflammatory insults that contribute to renal and systemic injury.

## References

[1] Schunk SJ, Zarbock A, Meersch M, Küllmar M, Kellum JA, Schmit D (2019). Association between urinary dickkopf-3, acute kidney injury, and subsequent loss of kidney function in patients undergoing cardiac surgery: an observational cohort study. Lancet.

[2] Cheruku SR, Raphael J, Neyra JA, Fox AA (2023). Acute kidney injury after cardiac surgery: prediction, prevention, and management. Anesthesiology.

[3] Lau D, Pannu N, James MT, Hemmelgarn BR, Kieser TM, Meyer SR (2021). Costs and consequences of acute kidney injury after cardiac surgery: a cohort study. J Thorac Cardiovasc Surg.

[4] Thiele RH, Isbell JM, Rosner MH (2015). AKI associated with cardiac surgery. Clin J Am Soc Nephrol.

[5] Wang Y, Bellomo R (2017). Cardiac surgery-associated acute kidney injury: risk factors, pathophysiology and treatment. Nat Rev Nephrol.

[6] Cuttone G, La Via L, SenussiTesta T, Sinatra N, Deana C, Roberti E (2026). Acute kidney injury in cardiac surgery: a comprehensive review of perioperative strategies and emerging biomarkers. J Cardiothorac Vasc Anesth.

[7] Prabhakar A, Ward CT, Tidwell AM, Angeles IW, Boorman DW, Ma J (2026). Cardiac surgery-associated acute kidney injury in a single healthcare system- a retrospective observational study. Crit Care Sci.

[8] Zarbock A, Weiss R, Albert F, Rutledge K, Kellum JA, Bellomo R (2023). EPIS-AKI Investigators. Epidemiology of surgery associated acute kidney injury (EPIS-AKI): a prospective international observational multi-center clinical study. Intensive Care Med.

[9] Strauß C, Albert F, Bormann E, Engelman DT, Bellomo R, Zarbock A, EPIS-AKI investigators (2026). Risk factors, outcomes, and early prediction of cardiac surgery-associated acute kidney injury: a post hoc subgroup analysis of the Epidemiology of Surgery Associated Acute Kidney Injury study. Br J Anaesth.

[10] Oosterom-Eijmael MJ, Hermanns H, Lankadeva YR, Hulst AH (2026). Cardiac surgery-associated acute kidney injury. BJA Educ.

[11] Scurt FG, Bose K, Mertens PR, Chatzikyrkou C, Herzog C (2024). Cardiac surgery-associated acute kidney injury. Kidney 360.

[12] Ortega-Loubon C, Tamayo E, Jorge-Monjas P (2022). Cardiac surgery-associated acute kidney injury: current updates and perspectives. J Clin Med.

